# Preferences for physician sex among adults attending Syrian public teaching hospitals: A multicenter cross-sectional study

**DOI:** 10.1371/journal.pone.0359066

**Published:** 2026-09-24

**Authors:** Ahmad Bishr Nasra, Malaka Abubakir, Lana Sabbagh, Zakaria Huseen, Mohammad Nasra, Mohammad Wesam Almohammad Alibrahim, Basel Zeitoun, Maria Kheder, Mohamad Morjan

**Affiliations:** 1 Faculty of Medicine, Damascus University, Damascus, Syrian Arab Republic; 2 Faculty of Medicine, University of Aleppo, Aleppo, Syrian Arab Republic; 3 Faculty of Medicine, University of Tartous, Tartous, Syrian Arab Republic; 4 Faculty of Human Medicine, University of Hama, Hama, Syrian Arab Republic; 5 Faculty of Medicine, Latakia University, Latakia, Syrian Arab Republic; 6 Department of Pediatric Surgery, Aleppo University Hospital, University of Aleppo, Aleppo, Syrian Arab Republic; Mayo Clinic Cancer Center, UNITED STATES OF AMERICA

## Abstract

**Background:**

Physician sex can influence communication, comfort, and care-seeking. Evidence from conservative contexts suggests preferences are strongest in intimate examinations and vary by specialty. Local data from Syria are limited.

**Methods:**

We conducted a multicenter cross-sectional survey in major Syrian public teaching hospitals. Adults attending participating hospitals, including patients and accompanying companions, completed an interviewer-administered questionnaire adapted from prior work and reviewed for clarity in Arabic, capturing preference for physician sex (male, female, no preference) across (i) attitudinal domains, (ii) common clinical interactions (e.g., history taking, general and intimate examinations, emergencies, procedures), and (iii) medical specialties. Demographics included age, participant sex, marital status, education, religion, religiosity, and income. Associations were assessed using chi-square tests and multinomial logistic regression with “no preference” as the reference category.

**Results:**

Across attitudinal domains and routine interactions, many participants reported no preference. In sensitive contexts, particularly intimate examinations, sex-concordant care was frequently preferred. Female participants tended to prefer female physicians for intimate and reproductive health encounters, whereas male participants more often preferred male physicians for general and intimate examinations. By specialty, preference for male physicians was more common in surgery-related fields (e.g., general surgery, orthopedics, urology), while preference for female physicians predominated in obstetrics/gynecology and select relational specialties (e.g., psychiatry, dermatology). In emergency or life-threatening scenarios, neutrality was common. Exploratory adjusted analyses identified several demographic associations, with participant sex showing the most consistent pattern across multiple contexts.

**Conclusions:**

Preferences for physician sex among adults attending Syrian public teaching hospitals were context-dependent, peaking in intimate examinations and varying by specialty. Ensuring feasible options for sex-concordant care in selected services, alongside communication training for all clinicians, may improve comfort and engagement among hospital attendees. Future research should evaluate how these preferences affect care utilization and outcomes, and explore clinician factors (e.g., seniority, communication style) that may mitigate or amplify preference effects.

## Introduction

The quality of the physician–patient relationship depends on clinical, personal, and socio-cultural factors; among these, physician sex can shape communication, perceived comfort, and willingness to seek care [1–3]. Physician sex has also been linked to differences in medical communication, while trust and shared decision-making are central elements of patient-centered clinical encounters [4,5]. Preferences are context-dependent: neutrality is common in routine care, but stronger sex-related preferences emerge in intimate examinations and specialties that involve bodily exposure or sensitive discussions, most notably in obstetrics and gynecology and, to a scenario-dependent extent, in urology [6,7].

Cultural and religious norms further influence these choices, including modesty considerations that can affect disclosure, acceptance of examination, and timing of care; studies from Muslim-majority and culturally conservative settings report heightened preference for sex-concordant care in sensitive encounters, sometimes with delays when concordance is unavailable [8]. At the same time, reviews note that for non-intimate scenarios many individuals prioritize clinician experience and reputation over physician sex, and evidence linking sex concordance to clinical outcomes is not uniform [2,9].

Despite growing global literature, empirical evidence from Syria is limited. Addressing this gap can inform service planning and education while respecting local norms. We therefore aimed to characterize preferences for physician sex among adults attending Syrian public teaching hospitals across common clinical interactions and specialties, and to examine demographic correlates of preferring a male physician, a female physician, or having no preference using a multicenter cross-sectional survey.

## Methods

### Study design and setting

We conducted a multicenter cross-sectional study between May 1 and June 15, 2025 in major public teaching hospitals across Syria. The objective was to assess adults’ preferences regarding physician sex in routine clinical encounters, with emphasis on physical examinations and procedure-related scenarios. Data were collected from seven public teaching hospitals across six recruitment governorates: Al-Mouwasat University Hospital and the National University Hospital in Damascus, Aleppo University Hospital, Latakia University Hospital, Hama National Hospital, Tartous National Hospital, and Idlib University Hospital. The recruitment governorates were Damascus (n = 342), Tartous (n = 204), Hama (n = 200), Latakia (n = 161), Aleppo (n = 142), and Idlib (n = 51).

### Participants and sampling

During the study period, eligible adults aged 18 years or older who were attending the participating hospitals were approached for participation. Eligible participants included patients and accompanying companions or family members. Healthcare workers and hospital staff were not included. Eligibility required capacity to provide verbal informed consent and Arabic proficiency sufficient for interview. Individuals younger than 18 years or declining participation were excluded. Recruitment was conducted in mixed hospital areas, including inpatient wards and other patient-care or waiting areas, using non-probability convenience sampling with consecutive invitation of eligible adults when feasible. The study was not restricted to a specific diagnosis, specialty, ward, or inpatient group. Participant role at the time of recruitment, reason for hospital attendance, current diagnosis, treating department, inpatient/outpatient status, and treating physician sex were not systematically recorded.

### Data collection procedures

Data were collected via structured, face-to-face interviews conducted by trained data collectors using a standardized script to minimize interviewer variability. Interviews were chosen to reduce literacy barriers, ensure comprehension, and allow clarification using Arabic wording that was understandable to participants. Interviews were conducted in locations that preserved auditory privacy as much as possible within the hospital setting, and each participant provided responses independently.

### Survey tool

The questionnaire comprised approximately 40 multiple-choice items organized into four sections:

1. Socio-demographics: age, participant sex, marital status, educational level, religion, religious commitment, and economic status.
2. Attitudinal domains: trait-specific physician-sex preference (male, female, no preference) regarding credibility, communication, listening, cooperation, and knowledge/expertise.
3. Clinical domains: physician-sex preference in common care contexts (e.g., medical history taking, discussion of psychological issues, general examination, genital examination, surgical procedures, life-threatening conditions). Two items, breast examination and labor/delivery, were administered to women only.
4. Medical specialties: physician-sex preference across named specialties (e.g., internal medicine, emergency medicine, obstetrics and gynecology, general surgery, ophthalmology, otolaryngology, urology, plastic surgery, dermatology, pediatrics, orthopedic surgery, psychiatry, neurology).

This questionnaire was adapted from a previously published tool developed by Alyahya et al. (2019) [10]. The questionnaire was translated into Arabic and reviewed by the research team for clarity, cultural appropriateness, and consistency with the original items. Because data were collected through face-to-face interviews, trained data collectors could clarify item wording when needed without changing the response options. No formal psychometric validation was performed in the Syrian population, and no separate pilot sample was excluded from the final analysis.

### Variables

For each trait, clinical domain, and specialty, the primary outcome was physician-sex preference with three categories: male physician preferred, female physician preferred, and no preference. For women-only items, breast examination and labor/delivery, analyses were restricted to female participants and participant sex was not included as a predictor.

Predictors included:

- Participant sex: male, female.
- Age: 18–25, 26–45, 46–59, ≥ 60 years.
- Marital status: single, currently in a relationship/married, previously married.
- Education: no formal or elementary education; middle to secondary education; bachelor’s degree or higher.
- Religion: Islam; Christianity.
- Religious commitment: weak; intermediate; strong.
- Economic status: low; intermediate or higher.

Analyses used complete cases; the final dataset comprised 1,100 valid responses.

### Sample size

The minimum required sample size was estimated as 384 participants, assuming a 50% expected prevalence of any physician-sex preference, 95% confidence level, and 5% margin of error. To allow subgroup analyses across demographic groups and preference domains, we aimed to recruit a larger sample. The final analytic sample included 1,100 valid responses from adults recruited across participating hospitals and governorates.

### Statistical analysis

Data were analyzed using IBM SPSS Statistics version 29.0 (IBM Corp., Armonk, NY). Descriptive statistics, including frequencies and percentages, summarized participants’ sociodemographic characteristics and physician-sex preferences. Associations between demographic variables and physician-sex preference were first examined using the chi-square test of independence. For significant chi-square results, post hoc adjusted standardized residuals were reviewed to identify specific subgroup differences. Residuals with absolute values ≥1.96 were considered statistically significant at p < 0.05. Given the large number of comparisons, bivariate analyses were interpreted as exploratory and used primarily to describe patterns across domains.

To assess independent predictors, multinomial logistic regression was conducted with physician-sex preference as the dependent variable, using “no preference” as the reference category. Independent variables included age, participant sex, marital status, educational level, religiosity, and economic status. For female-specific domains, breast examination and labor/delivery, participant sex was excluded as a predictor. The highest categories, including age ≥ 60 years, previously married, bachelor’s degree or higher, strong religiosity, and intermediate or higher economic status, were designated as reference groups. Results are presented as adjusted relative risk ratios (aRRR) with 95% confidence intervals (CI). Model fit was evaluated using Pearson and deviance statistics, pseudo-R^2^ indices (Cox and Snell, Nagelkerke, and McFadden), and likelihood ratio tests. Given the number of domain-specific regression models and predictor contrasts, the regression analyses were considered exploratory. No formal adjustment for multiple testing was applied. Accordingly, isolated statistically significant associations, particularly those near p = 0.05 or accompanied by wide confidence intervals, were interpreted cautiously, with greater emphasis on consistency across domains, effect sizes, and confidence intervals.

### Ethical approvals

Ethical approval was obtained from the Faculty of Medicine at Aleppo University (Approval ID: 2527), and formal permission was obtained from the Syrian Ministry of Health and/or participating public hospitals as required for data collection in public hospital settings. All responses were collected anonymously, and no personal identifying information was recorded. Participant privacy was maintained throughout data collection. The study followed the ethical principles of the Declaration of Helsinki. Participants received a clear explanation of the study and its objectives, and verbal informed consent was obtained before beginning each interview.

## Results

### Demographic characteristics

Of the 1,100 participants, 572 (52.0%) were women and 528 (48.0%) were men. Participants were recruited from six governorates: Damascus (342, 31.1%), Tartous (204, 18.5%), Hama (200, 18.2%), Latakia (161, 14.6%), Aleppo (142, 12.9%), and Idlib (51, 4.6%). The largest age group was 26–45 years (37.3%), followed by 18–25 years (26.7%), 46–59 years (24.7%), and ≥60 years (11.3%). Most participants were currently in a relationship/married (63.1%), while 28.9% were single and 8.0% were previously married. Nearly half of the sample (46.1%) had attained a bachelor’s degree or higher, while 33.3% had middle to secondary education, and 20.6% had no formal or elementary education. The majority of respondents were Muslim (96.5%), with a small minority identifying as Christian (3.5%). Levels of religious commitment varied, with 53.5% reporting intermediate commitment, 37.5% strong commitment, and 9.0% weak commitment. Over one-third of participants (36.9%) reported low economic status, while 63.1% reported intermediate or higher status (Table 1).

**Table 1:** Sociodemographic characteristics of participants by participant sex.

Variable	Category	Women (n = 572)	Men (n = 528)	Total (n = 1,100)
Recruitment governorate	Damascus	172 (30.1%)	170 (32.2%)	342 (31.1%)
	Tartous	122 (21.3%)	82 (15.5%)	204 (18.5%)
	Hama	111 (19.4%)	89 (16.9%)	200 (18.2%)
	Latakia	64 (11.2%)	97 (18.4%)	161 (14.6%)
	Aleppo	82 (14.3%)	60 (11.4%)	142 (12.9%)
	Idlib	21 (3.7%)	30 (5.7%)	51 (4.6%)
Age	18–25	151 (26.4%)	143 (27.1%)	294 (26.7%)
	26–45	238 (41.6%)	172 (32.6%)	410 (37.3%)
	46–59	143 (25.0%)	129 (24.4%)	272 (24.7%)
	≥60	40 (7.0%)	84 (15.9%)	124 (11.3%)
Marital status	Single	149 (26.0%)	169 (32.0%)	318 (28.9%)
	Currently in a relationship/married	366 (64.0%)	328 (62.1%)	694 (63.1%)
	Previously married	57 (10.0%)	31 (5.9%)	88 (8.0%)
Educational level	No formal or elementary education	137 (24.0%)	90 (17.0%)	227 (20.6%)
	Middle to secondary education	162 (28.3%)	204 (38.6%)	366 (33.3%)
	Bachelor’s degree or higher	273 (47.7%)	234 (44.3%)	507 (46.1%)
Religion	Islam	547 (95.6%)	514 (97.3%)	1,061 (96.5%)
	Christianity	25 (4.4%)	14 (2.7%)	39 (3.5%)
Religious commitment	Weak	45 (7.9%)	54 (10.2%)	99 (9.0%)
	Intermediate	293 (51.2%)	296 (56.1%)	589 (53.5%)
	Strong	234 (40.9%)	178 (33.7%)	412 (37.5%)
Economic status	Low	203 (35.5%)	203 (38.4%)	406 (36.9%)
	Intermediate or higher	369 (64.5%)	325 (61.6%)	694 (63.1%)

### Physician attitude and professional competence sex preferences

Participants’ preferences for physician sex varied across professional traits. For credibility, knowledge and expertise, and understanding of psychological needs, most participants reported no preference, with only minor differences between men and women (p > 0.05). By contrast, significant participant-sex differences were observed in preferences related to communication (p < 0.001), cooperation (p < 0.001), and listening to patients (p = 0.006). Male participants were more likely than female participants to prefer male physicians for these traits, while women more often expressed no preference or favored female physicians (Table 2).

**Table 2:** Physician-sex preferences based on physician attitude and professional competence domains.

Factor	Participant sex	Male physician preferred n (%)	Female physician preferred n (%)	No preference n (%)	p-value
Credibility	Male	163 (30.9%)	51 (9.7%)	314 (59.5%)	0.069
	Female	141 (24.7%)	58 (10.1%)	373 (65.2%)	
Understanding of psychological needs	Male	123 (23.3%)	173 (32.8%)	232 (43.9%)	0.112
	Female	107 (18.7%)	184 (32.2%)	281 (49.1%)	
Medical knowledge and expertise	Male	236 (44.7%)	32 (6.1%)	260 (49.2%)	0.177
	Female	224 (39.2%)	39 (6.8%)	309 (54.0%)	
Communication	Male	201 (38.1%)	126 (23.9%)	201 (38.1%)	<0.001
	Female	146 (25.5%)	158 (27.6%)	268 (46.9%)	
Cooperation	Male	165 (31.3%)	136 (25.8%)	227 (43.0%)	<0.001
	Female	121 (21.2%)	129 (22.6%)	322 (56.3%)	
Listening to the patient	Male	154 (29.2%)	158 (29.9%)	216 (40.9%)	0.006
	Female	139 (24.3%)	144 (25.2%)	289 (50.5%)	

### Other demographic variables

Chi-square analyses demonstrated multiple significant associations between participants’ demographic characteristics and their preferences for physician sex across professional traits. Given the large number of bivariate comparisons, these findings should be interpreted as exploratory. Age was significantly associated with all six traits (p ≤ 0.025), while marital status showed strong associations with credibility, understanding of psychological needs, communication, cooperation, and listening (all p ≤ 0.016). Educational level was significantly linked to psychological understanding, medical knowledge and expertise, communication, cooperation, and listening (all p ≤ 0.014). Religion showed an association only with credibility (p = 0.041). Religiosity was strongly associated with listening (p < 0.001), and income was not significantly related to preferences in any trait (Table 3).

**Table 3:** Association between demographic variables and physician-sex preference across competence domains.

Factor	Age	Marital status	Education	Religion	Religiosity	Income
Credibility	0.002	0.010	0.091	0.041	0.654	0.911
Understanding of psychological needs	<0.001	0.001	<0.001	0.760	0.066	0.210
Medical knowledge and expertise	<0.001	0.052	<0.001	0.363	0.094	0.134
Communication	<0.001	0.016	<0.001	0.096	0.164	0.764
Cooperation	0.025	0.004	0.014	0.708	0.063	0.526
Listening to the patient	<0.001	<0.001	0.001	0.781	<0.001	0.697

### Preference distribution across specific clinical scenarios

Clear participant-sex differences were observed across all clinical domains. Men consistently demonstrated a strong preference for male physicians, particularly in sensitive procedures such as general examinations (66.9% vs. 18.9% in women), genital examinations (76.3% vs. 6.5%), and surgical procedures (66.7% vs. 49.0%) (all p < 0.001). By contrast, women were more likely to prefer female physicians, especially for psychological issues (43.2% vs. 24.1% in men), general examination (47.7% vs. 5.7%), genital examination (71.9% vs. 4.7%), and surgical procedures (11.7% vs. 1.3%).

For female-specific domains, the majority of women preferred female physicians for both breast examination (73.6%) and labor and delivery (63.1%). In urgent scenarios, such as life-threatening conditions, both sexes leaned toward neutrality, though men remained more likely to prefer male physicians (45.5% vs. 33.2%) (Table 4).

**Table 4:** Physician-sex preference distribution across specific clinical scenarios.

Factor	Participant sex	Male physician preferred n (%)	Female physician preferred n (%)	No preference n (%)	p-value
Medical history taking	Male	229 (43.4%)	69 (13.1%)	230 (43.6%)	<0.001
	Female	132 (23.1%)	133 (23.3%)	307 (53.7%)	
Discussion of psychological issues	Male	225 (42.6%)	127 (24.1%)	176 (33.3%)	<0.001
	Female	110 (19.2%)	247 (43.2%)	215 (37.6%)	
General examination	Male	353 (66.9%)	30 (5.7%)	145 (27.5%)	<0.001
	Female	108 (18.9%)	273 (47.7%)	191 (33.4%)	
Genital examination	Male	403 (76.3%)	25 (4.7%)	100 (18.9%)	<0.001
	Female	37 (6.5%)	411 (71.9%)	124 (21.7%)	
Breast examination	Female only	26 (4.5%)	421 (73.6%)	125 (21.9%)	—
Life-threatening conditions	Male	240 (45.5%)	19 (3.6%)	269 (50.9%)	<0.001
	Female	190 (33.2%)	47 (8.2%)	335 (58.6%)	
Surgical procedures	Male	352 (66.7%)	7 (1.3%)	169 (32.0%)	<0.001
	Female	280 (49.0%)	67 (11.7%)	225 (39.3%)	
Labor and delivery	Female only	55 (9.6%)	361 (63.1%)	156 (27.3%)	—

### Other demographic variables

Significant associations were identified between multiple demographic variables and physician-sex preferences in clinical domains. These bivariate findings should be interpreted as exploratory. Age was associated with history taking, psychological issues, general examination, and genital examination (all p < 0.001). Marital status was associated with medical history taking, general examination, genital examination, and surgical procedures (p ≤ 0.036). Educational level was significantly related to medical history taking, psychological issues, general examination, genital examination, breast examination, life-threatening conditions, surgical procedures, and labor and delivery (p ≤ 0.033).

Smaller but notable effects were observed for religion, which was associated with genital examination (p = 0.013) and labor and delivery (p = 0.004). Religiosity was linked to genital examination (p = 0.014), breast examination (p = 0.034), surgical procedures (p = 0.047), and labor and delivery (p = 0.002). In contrast, income was not significantly associated with preferences in any of the clinical domains (Table 5).

**Table 5:** Associations between demographics and physician-sex preference across specific clinical scenarios.

Procedure	Age	Marital status	Education	Religion	Religiosity	Income
Medical history taking	<0.001	0.036	0.004	0.513	0.647	0.464
Discussion of psychological issues	<0.001	0.465	0.004	0.946	0.218	0.260
General examination	<0.001	0.001	<0.001	0.174	0.471	0.257
Genital examination	<0.001	0.001	<0.001	0.013	0.014	0.658
Breast examination	0.795	0.110	0.033	0.455	0.034	0.648
Life-threatening conditions	0.637	0.154	0.018	0.542	0.097	0.109
Surgical procedures	0.140	0.013	0.003	0.284	0.047	0.121
Labor and delivery	0.408	0.063	0.009	0.004	0.002	0.322

### Preference by medical specialty

Clear participant-sex-based trends emerged across medical specialties. Male participants expressed stronger preferences for male physicians in fields involving surgery or urology, such as general surgery (67.4%), orthopedic surgery (69.5%), and urology (75.9%). Conversely, female participants were more likely to prefer female physicians in obstetrics and gynecology (65.0%), urology (44.6%), dermatology (35.3%), and psychiatry (25.3%). Several specialties, including ophthalmology and plastic surgery, exhibited a large proportion of no preference. Otolaryngology showed a small but significant difference (p = 0.029), and emergency medicine displayed stronger male physician preference in both sexes, though more pronounced among males (55.7%). These variations indicate that physician-sex preferences differed by specialty and by participant sex (Table 6).

**Table 6:** Participants’ physician-sex preferences according to medical specialty.

Specialty	Participant sex	Male physician preferred n (%)	Female physician preferred n (%)	No preference n (%)	p-value
Internal medicine	Male	251 (47.5%)	28 (5.3%)	249 (47.2%)	<0.001
	Female	147 (25.7%)	97 (17.0%)	328 (57.3%)	
Emergency medicine	Male	294 (55.7%)	19 (3.6%)	215 (40.7%)	<0.001
	Female	213 (37.2%)	41 (7.2%)	318 (55.6%)	
Obstetrics and gynecology	Male	44 (8.3%)	215 (40.7%)	269 (51.0%)	<0.001
	Female	67 (11.7%)	372 (65.0%)	133 (23.3%)	
General surgery	Male	356 (67.4%)	13 (2.5%)	159 (30.1%)	<0.001
	Female	293 (51.2%)	51 (8.9%)	228 (39.9%)	
Ophthalmology	Male	184 (34.8%)	50 (9.5%)	294 (55.7%)	0.701
	Female	196 (34.3%)	63 (11.0%)	313 (54.7%)	
Otolaryngology	Male	181 (34.3%)	42 (8.0%)	305 (57.8%)	0.029
	Female	166 (29.0%)	69 (12.1%)	337 (58.9%)	
Urology	Male	401 (75.9%)	14 (2.7%)	113 (21.4%)	<0.001
	Female	146 (25.5%)	255 (44.6%)	171 (29.9%)	
Plastic surgery	Male	166 (31.4%)	124 (23.5%)	238 (45.1%)	0.693
	Female	176 (30.8%)	147 (25.7%)	249 (43.5%)	
Dermatology	Male	145 (27.5%)	108 (20.5%)	275 (52.1%)	<0.001
	Female	100 (17.5%)	202 (35.3%)	270 (47.2%)	
Pediatrics	Male	125 (23.7%)	148 (28.0%)	255 (48.3%)	0.036
	Female	167 (29.2%)	128 (22.4%)	277 (48.4%)	
Orthopedic surgery	Male	367 (69.5%)	8 (1.5%)	153 (29.0%)	<0.001
	Female	328 (57.3%)	25 (4.4%)	219 (38.3%)	
Psychiatry	Male	176 (33.3%)	105 (19.9%)	247 (46.8%)	<0.001
	Female	125 (21.9%)	145 (25.3%)	302 (52.8%)	
Neurology	Male	260 (49.2%)	30 (5.7%)	238 (45.1%)	0.003
	Female	227 (39.7%)	51 (8.9%)	294 (51.4%)	

### Other demographic variables

Table 7 presents the p-values reflecting associations between participants’ demographic characteristics and their physician-sex preferences across various medical specialties. These bivariate findings should be interpreted as exploratory. Age showed significant associations with preferences in obstetrics and gynecology, general surgery, ophthalmology, otolaryngology, urology, dermatology, pediatrics, and psychiatry. Marital status was significantly related to preferences in internal medicine, general surgery, ophthalmology, otolaryngology, urology, plastic surgery, dermatology, pediatrics, and psychiatry. Educational level was associated with preferences in several specialties, including internal medicine, general surgery, ophthalmology, otolaryngology, urology, plastic surgery, dermatology, pediatrics, psychiatry, and neurology. Religion showed significance only in obstetrics and gynecology, while religiosity was associated with urology, dermatology, pediatrics, psychiatry, and neurology. Income level had limited influence, with a statistically significant association observed only in emergency medicine (Table 7).

**Table 7:** Association between participants’ demographics and physician-sex preference across medical specialties.

Specialty	Age	Marital status	Education	Religion	Religiosity	Income
Internal medicine	0.066	0.033	<0.001	0.759	0.082	0.366
Emergency medicine	0.305	0.263	0.064	0.996	0.264	0.029
Obstetrics and gynecology	0.001	0.647	0.053	0.028	0.098	0.242
General surgery	0.033	0.019	0.005	0.141	0.456	0.499
Ophthalmology	<0.001	<0.001	0.015	0.867	0.109	0.804
Otolaryngology	<0.001	<0.001	<0.001	0.561	0.336	0.863
Urology	0.033	0.038	0.029	0.054	0.043	0.789
Plastic surgery	0.444	0.020	<0.001	0.203	0.298	0.469
Dermatology	<0.001	0.003	<0.001	0.179	0.037	0.065
Pediatrics	<0.001	<0.001	<0.001	0.667	0.001	0.111
Orthopedic surgery	0.463	0.527	0.638	0.191	0.628	0.282
Psychiatry	<0.001	0.030	<0.001	0.236	0.039	0.171
Neurology	0.072	0.374	0.015	0.468	0.001	0.583

## Regression results by clinical domain

### Model specification

Multinomial logistic regression models were fitted separately for medical history taking, psychological issues, general examination, genital examination, surgical procedures, and life-threatening conditions. Outcomes were physician-sex preference (male physician preferred, female physician preferred, no preference; reference = no preference). Predictors included participant sex, age, marital status, education, religiosity, and income, with reference categories set as: female participant sex, ≥ 60 years, previously married, bachelor’s degree or higher, strong religiosity, and intermediate or higher income (Table 8). Given their exploratory nature and the absence of multiplicity adjustment, isolated associations from these models should be interpreted cautiously; emphasis is placed on consistency, effect sizes, and confidence intervals.

**Table 8:** Multinomial logistic regression of demographic predictors of physician-sex preference across six clinical domains.

Domain and comparison	Predictor	aRRR	95% CI	p-value
Taking medical history: Male preference vs no preference	Male participant vs female participant	2.25	1.69–2.99	<0.001
	Middle to secondary education vs bachelor’s degree or higher	1.42	1.02–1.97	0.036
Taking medical history: Female preference vs no preference	Male participant vs female participant	0.68	0.48–0.97	0.033
Discussion of psychological issues: Male preference vs no preference	Male participant vs female participant	2.70	1.96–3.70	<0.001
	No formal or elementary education vs bachelor’s degree or higher	1.97	1.29–3.01	0.002
	Intermediate vs strong religiosity	0.71	0.51–0.98	0.039
Discussion of psychological issues: Female preference vs no preference	Male participant vs female participant	0.66	0.49–0.90	0.008
	Age 18–25 vs ≥ 60 years	2.32	1.22–4.42	0.010
	Age 26–45 vs ≥ 60 years	2.22	1.27–3.86	0.005
General examination: Male preference vs no preference	Male participant vs female participant	4.28	3.10–5.89	<0.001
	Middle to secondary education vs bachelor’s degree or higher	1.63	1.13–2.34	0.009
General examination: Female preference vs no preference	Male participant vs female participant	0.14	0.09–0.22	<0.001
	Middle to secondary education vs bachelor’s degree or higher	1.83	1.20–2.78	0.005
Genital examination: Male preference vs no preference	Male participant vs female participant	13.84	8.87–21.61	<0.001
Genital examination: Female preference vs no preference	Male participant vs female participant	0.07	0.05–0.12	<0.001
	Middle to secondary education vs bachelor’s degree or higher	1.64	1.03–2.62	0.036
	Intermediate vs strong religiosity	0.66	0.44–0.98	0.039
Surgical procedures: Male preference vs no preference	Male participant vs female participant	1.71	1.31–2.23	<0.001
	Intermediate vs strong religiosity	0.69	0.52–0.92	0.012
Surgical procedures: Female preference vs no preference	Male participant vs female participant	0.13	0.06–0.30	<0.001
	Single vs previously married	9.18	1.07–78.70	0.043
	Currently in a relationship/married vs previously married	13.30	1.75–101.25	0.012
	No formal or elementary education vs bachelor’s degree or higher	2.60	1.27–5.32	0.009
	Middle to secondary education vs bachelor’s degree or higher	2.06	1.03–4.11	0.040
Life-threatening conditions: Male preference vs no preference	Male participant vs female participant	1.66	1.28–2.16	<0.001
	Single vs previously married	0.51	0.29–0.91	0.022
Life-threatening conditions: Female preference vs no preference	Male participant vs female participant	0.46	0.26–0.82	0.009
	No formal or elementary education vs bachelor’s degree or higher	2.90	1.39–6.03	0.004
	Middle to secondary education vs bachelor’s degree or higher	2.60	1.33–5.09	0.005
	Low vs intermediate/higher income	0.53	0.29–0.94	0.029

### Participant sex

Participant sex was the strongest and most consistent predictor across all domains. Men were more likely than women to prefer male physicians in history taking (aRRR = 2.25, 95% CI: 1.69–2.99, p < 0.001), psychological discussions (aRRR = 2.70, 95% CI: 1.96–3.70, p < 0.001), general examinations (aRRR = 4.28, 95% CI: 3.10–5.89, p < 0.001), genital examinations (aRRR = 13.84, 95% CI: 8.87–21.61, p < 0.001), surgical procedures (aRRR = 1.71, 95% CI: 1.31–2.23, p < 0.001), and life-threatening conditions (aRRR = 1.66, 95% CI: 1.28–2.16, p < 0.001). Conversely, men were significantly less likely than women to prefer female physicians in these same domains, with the strongest effect in genital examinations (aRRR = 0.07, 95% CI: 0.05–0.12, p < 0.001).

### Education

Education showed several exploratory associations. Middle to secondary education was associated with greater male preference in history taking (aRRR = 1.42, p = 0.036) and general examination (aRRR = 1.63, p = 0.009). It was also associated with greater female preference in general examination (aRRR = 1.83, p = 0.005), genital examination (aRRR = 1.64, p = 0.036), surgical procedures (aRRR = 2.06, p = 0.040), and life-threatening conditions (aRRR = 2.60, p = 0.005). No formal or elementary education was associated with greater male preference in psychological issues (aRRR = 1.97, p = 0.002) and greater female preference in surgical procedures (aRRR = 2.60, p = 0.009) and life-threatening conditions (aRRR = 2.90, p = 0.004).

### Age

Age showed domain-specific exploratory associations. In psychological discussions, younger participants aged 18–25 and 26–45 years were more than twice as likely as those aged ≥60 years to prefer female physicians (aRRR = 2.32, p = 0.010; aRRR = 2.22, p = 0.005, respectively). No other domains showed consistent age associations.

### Marital status

Marital status showed associations in selected domains. Single participants were less likely to prefer male physicians in life-threatening conditions (aRRR = 0.51, p = 0.022). In contrast, both single and currently in a relationship/married participants were more likely than previously married participants to prefer female physicians for surgical procedures (aRRR = 9.18, p = 0.043; aRRR = 13.30, p = 0.012). These surgical-procedure estimates had wide confidence intervals and should be interpreted cautiously.

### Religiosity

Religiosity was associated with selected domains. Intermediate religiosity was associated with lower male preference in psychological issues (aRRR = 0.71, p = 0.039) and surgical procedures (aRRR = 0.69, p = 0.012). It was also associated with lower female preference in genital examinations (aRRR = 0.66, p = 0.039).

### Income

Income associations were less consistent. Low income was associated with lower female preference in life-threatening conditions (aRRR = 0.53, p = 0.029). No statistically significant associations were observed in other domains.

## Breast examination and labor/delivery

### Model specification

Separate models were fitted for breast examination and labor/delivery. These domains were restricted to female participants only; therefore, participant sex was excluded as a predictor. Predictors included age, marital status, education, religiosity, and income, with reference groups as above (Table 9).

**Table 9:** Multinomial logistic regression of demographic predictors of physician-sex preference in female-specific clinical domains.

Domain and comparison	Predictor	aRRR	95% CI	p-value
Breast examination: Male preference vs no preference	No formal or elementary education vs bachelor’s degree or higher	3.86	1.14–13.09	0.030
Breast examination: Female preference vs no preference	Currently in a relationship/married vs previously married	2.24	1.18–4.25	0.014
	Weak vs strong religiosity	0.44	0.21–0.93	0.031
	Intermediate vs strong religiosity	0.60	0.37–0.95	0.029
Labor/delivery: Female preference vs no preference	Age 18–25 vs ≥ 60 years	2.62	1.04–6.59	0.041
	No formal or elementary education vs bachelor’s degree or higher	1.95	1.11–3.45	0.021
	Middle to secondary education vs bachelor’s degree or higher	1.89	1.13–3.17	0.015
	Weak vs strong religiosity	0.30	0.15–0.62	0.001
	Intermediate vs strong religiosity	0.64	0.41–0.99	0.046

### Breast examination

Women with no formal or elementary education were more likely than those with a bachelor’s degree or higher to prefer male physicians (aRRR = 3.86, p = 0.030). Currently in a relationship/married women were more likely than previously married women to prefer female physicians (aRRR = 2.24, p = 0.014). Weak religiosity (aRRR = 0.44, p = 0.031) and intermediate religiosity (aRRR = 0.60, p = 0.029) were associated with lower preference for female physicians. Age and income were not significant predictors.

### Labor and delivery

Younger women aged 18–25 years were more likely than those aged ≥60 years to prefer female physicians (aRRR = 2.62, p = 0.041). No formal or elementary education (aRRR = 1.95, p = 0.021) and middle to secondary education (aRRR = 1.89, p = 0.015) were associated with greater female preference compared with bachelor’s degree or higher. Weak religiosity (aRRR = 0.30, p = 0.001) and intermediate religiosity (aRRR = 0.64, p = 0.046) were associated with lower preference for female physicians. Marital status and income were not significantly associated with preferences.

### Model fit and diagnostics

Model-fit statistics are summarized in Table 10. Pearson goodness-of-fit tests did not indicate poor fit for the adjusted models. The deviance goodness-of-fit test was significant only for the discussion of psychological issues model (p = 0.017), although the Pearson test for that model was not significant (p = 0.334). This discrepancy indicates possible model misfit and limits confidence in adjusted estimates from the psychological-issues model; those results should therefore be considered exploratory and interpreted with particular caution. Other models did not show statistically significant deviance misfit. Pseudo-R² values were highest for genital examination and general examination, consistent with the stronger role of participant sex in these domains. SPSS outputs indicated sparse subpopulation cells across models; therefore, isolated estimates with wide confidence intervals, particularly in the surgical-procedure model, should be interpreted cautiously.

**Table 10:** Model-fit summary for multinomial logistic regression models.

Model	Pearson goodness-of-fit p-value	Deviance goodness-of-fit p-value	Nagelkerke pseudo-R²
Taking medical history	0.591	0.148	0.092
Discussion of psychological issues	0.334	0.017	0.124
General examination	0.819	0.508	0.351
Genital examination	0.390	0.938	0.587
Surgical procedures	0.453	0.889	0.122
Life-threatening conditions	0.222	0.758	0.069
Breast examination	0.872	0.970	0.085
Labor/delivery	0.220	0.070	0.082

## Discussion

This study found that preferences for physician sex among adults attending Syrian public teaching hospitals are context-dependent, shaped by the type of medical interaction, and associated with demographic factors including participant sex, age, marital status, education, religiosity, income, medical specialty, and type of medical interaction. Overall, no preference was common in several routine or general domains, whereas preferences became more pronounced in intimate examinations and selected specialties. In exploratory adjusted analyses, participant sex was the most consistent predictor across clinical domains.

### Attitudinal domains

In our study, preferences related to general physician traits, such as credibility, knowledge, communication, cooperation, and listening, were influenced by demographic factors, although these effects were less pronounced than those observed in specific clinical interactions. Most participants reported no preference for credibility, understanding of psychological needs, and medical knowledge and expertise. Female participants tended to report no preference or to prefer female physicians for communication and listening, while male participants showed a stronger preference for male physicians in selected domains.

These findings are broadly consistent with previous literature. Roter et al. reported sex-related differences in medical communication, with female physicians generally showing more patient-centered communication patterns [3]. Schmid et al. [11] similarly reported that female patients may express higher satisfaction with female physicians who adopt a caring, sex-concordant communication style, and other work has examined differences in patient satisfaction with male versus female physicians [12]. However, our findings should not be interpreted as evidence that one physician sex is inherently more competent or communicative. Rather, they reflect participants’ stated preferences and expectations in a specific social and healthcare context.

Education also emerged as an important moderator in our data. Participants with a bachelor’s degree or higher tended to show more neutral responses in several domains, whereas participants with lower educational attainment demonstrated stronger physician-sex preferences in selected contexts. Age differences were also present, with younger participants showing more preference for female physicians in some communication-related domains, while older groups more often preferred male physicians in selected traits. These findings suggest that physician-sex preferences may vary across educational and generational groups. They are also compatible with earlier work showing that expectations of physician behavior can be related to preference for a female or male general practitioner [13].

Religiosity further shaped preferences in selected domains. Because our study did not ask participants why they preferred physicians of a specific sex, these associations should be interpreted cautiously. They may reflect differences in modesty concerns, social expectations, or prior experiences with healthcare, but the present cross-sectional survey cannot determine these mechanisms. Recognizing these influences may help guide more culturally sensitive physician–patient communication strategies while avoiding assumptions about individual patients’ preferences.

### Clinical interactions

Preferences for physician sex varied considerably depending on the type of clinical interaction. For medical history taking, both participant sex and education played important roles. Male participants were more than twice as likely as female participants to prefer male physicians, while middle to secondary education increased the likelihood of preferring male physicians. This differs from Saudi findings in which many patients expressed no strong physician-sex preference for history taking, although women showed some inclination toward female physicians [10]. These differences may reflect variation in setting, sampling frame, and social norms, rather than culture alone.

Psychological consultations revealed a different pattern. Participant sex, age, and education showed the main adjusted associations: men preferred male physicians, younger participants favored female physicians, and no formal or elementary education was linked to stronger male preference. Because the deviance goodness-of-fit test for this model was significant (p = 0.017), these adjusted associations warrant particular caution and should not be regarded as definitive. These observations are partly consistent with findings from Saudi Arabia [10], where participant sex influenced preferences in selected settings. The preference for female physicians in psychological discussions among younger participants may also align with broader literature linking physician sex and communication style, although our survey did not directly measure communication quality or empathy [3].

For general physical examinations, participant sex and education again emerged as the primary factors. Men strongly favored male physicians, while women were either neutral or leaned toward female physicians. This pattern aligns with Saudi findings [14], but differs from Brazilian data, where physician-sex preference was generally weaker [15]. This contrast suggests that preferences for physician sex may be more pronounced in settings where modesty norms, family expectations, or sex-segregated social experiences remain influential.

Preferences became highly polarized in genital examinations. Male participants overwhelmingly preferred male physicians, while women strongly favored female physicians. Comparable patterns were observed in Saudi Arabia [10,14], whereas Brazilian patients showed weaker sex-specific preferences overall, with stronger preferences mainly in specialties involving intimate examinations [15]. This supports the view that intimate exposure is a key context in which physician-sex preference becomes clinically relevant. In practice, this means that availability of both male and female clinicians, appropriate privacy, and respectful explanation before intimate examinations may be particularly important.

Life-threatening conditions showed a more neutral pattern. Although men still preferred male physicians more often than women did, no preference was the most common response among both sexes. This suggests that urgency may reduce, but does not fully eliminate, physician-sex preferences. Saudi studies have reported strong same-sex preferences in sensitive clinical contexts [14], whereas studies from Western emergency settings often report weaker physician-sex preferences [16]. These differences may reflect both cultural expectations and the way emergency care is organized and experienced across healthcare systems.

Surgical procedures further underscored the complexity of these preferences. Male participants tended to prefer male surgeons, while female preference was uncommon overall but more frequent among women than men. Education, marital status, and religiosity showed domain-specific associations. However, some adjusted estimates for marital status in the surgical model had wide confidence intervals, indicating statistical instability and the need for cautious interpretation. Similar specialty-level patterns have been documented in Saudi Arabia [14]. Therefore, physician sex may matter for some participants, but it should not be viewed as a substitute for perceived competence, professionalism, and trust.

Among women specifically, breast examinations and labor/delivery highlighted some of the strongest preferences for female physicians. Most women preferred female physicians for both breast examination and labor/delivery. This is consistent with literature showing stronger preferences for female clinicians during intimate or reproductive care [9,15]. At the same time, not all women preferred female physicians, and a meaningful minority reported no preference. This reinforces the importance of asking patients about preferences rather than assuming them based on sex, religiosity, or marital status.

Looking across specialties, our results showed that physician-sex preferences often clustered by perceived sensitivity and specialty type. Female physicians were strongly favored in obstetrics and gynecology and were also more often preferred by women in urology, dermatology, and psychiatry. Male physicians were more often preferred in general surgery, orthopedic surgery, urology among men, emergency medicine, and neurology. Some fields, including ophthalmology and plastic surgery, elicited little participant-sex difference. These patterns resemble findings from Saudi Arabia [14] and differ from Brazilian data, where most patients reported no general physician-sex preference and same-sex preference was more concentrated in intimate specialties [15].

Together, these results suggest that preferences for physician sex in Syria are not uniform. They are strongest in intimate examinations and selected specialties, but weaker or absent in many routine and non-intimate contexts. This distinction is important. It supports targeted accommodation of preferences where they are most clinically relevant, rather than broad assumptions that physician sex matters equally across all encounters.

### Interpretation

Overall, the findings suggest a context-specific pattern rather than a generalized preference for one physician sex. Stronger preferences in intimate and reproductive contexts may reflect modesty concerns, social norms, family expectations, prior experiences, or perceived comfort. However, because the study did not measure reasons for preferences, these explanations remain interpretive.

Several demographic factors appear to reinforce these patterns. Participant sex was the clearest factor, particularly in general and genital examinations. Education also played an important role, with higher education often associated with more neutral preferences, possibly because more educated participants may place greater emphasis on professional competence. Religiosity was associated with preferences in selected sensitive domains, but the direction and strength of this relationship varied. This variation shows that religiosity should not be treated as a simple or uniform predictor of physician-sex preference.

At the specialty level, preferences appear to reflect both clinical sensitivity and social perceptions of different fields. Obstetrics and gynecology and urology are directly connected to intimate or reproductive care, which may explain stronger sex-concordant preferences. Surgery and orthopedics may be shaped by perceptions of technical expertise or physical demands, but these interpretations require caution because the survey did not directly assess perceived competence, strength, or previous experiences with surgeons. Future qualitative work is needed to understand why participants associate specific specialties with physicians of a particular sex.

### Implications

The findings have practical implications for healthcare delivery in Syria and similar settings. First, hospitals should consider feasible ways to accommodate physician-sex preferences for intimate examinations, breast examination, and obstetric care when staffing allows. This should be implemented as a patient-centered option, not as a rigid rule. Second, clinicians of all sexes should receive training in privacy-preserving examination techniques, clear consent, respectful communication, and management of patient discomfort. These steps may reduce anxiety even when a sex-concordant physician is unavailable.

Third, service planning should avoid reinforcing stereotypes. The finding that some participants preferred male physicians in procedural specialties should not be used to limit opportunities for female physicians in surgery or other technical fields. Instead, institutions should promote visible competence, professionalism, and communication skills among all physicians. This is particularly important in regional settings where perceptions of female physicians and gender disparity in medicine have been documented [17]. Similarly, the preference for female physicians in obstetrics and intimate care should support patient comfort and access while preserving equal professional opportunities.

At the organizational level, flexible staffing, chaperone availability, clear privacy procedures, and respectful communication may help align care with patient comfort. Accommodating preferences is most defensible when it protects dignity, facilitates examination, or improves access to care. It should not compromise timely care, emergency treatment, or equitable workforce development.

### Limitations

This study has several limitations. First, data were collected through interviewer-administered questionnaires, which may have introduced social desirability bias. Participants may have underreported or overreported sensitive views related to religiosity, marital status, or physician-sex preferences. Although interviews were conducted with attention to privacy, complete privacy may not always have been possible in busy hospital settings.

Second, the study sample consisted of adults attending participating public teaching hospitals, including patients and accompanying companions or family members. Participant role at the time of recruitment was not systematically recorded. Therefore, the findings should be interpreted as preferences among adult hospital attendees rather than preferences among a clinically defined patient population. We also did not record reason for hospital attendance, current diagnosis, treating department, inpatient/outpatient status, or treating physician sex. As a result, we could not assess whether immediate clinical context or prior/current physician exposure influenced preferences.

Third, the sample was not fully representative of all Syrian adults. Data were collected from selected public teaching hospitals, and private hospitals, rural facilities, and adults not attending hospitals were not included. Certain governorates and minority groups were underrepresented. Therefore, generalizability beyond adults attending these public hospital settings should be made cautiously.

Fourth, the cross-sectional design prevents causal inference. The study identifies associations between demographic factors and physician-sex preferences, but it cannot determine directionality or causality. Previous experiences with physicians, whether positive or negative, may have influenced preferences but were not measured.

Fifth, the questionnaire was adapted from previous work and reviewed for Arabic clarity and cultural appropriateness, but formal psychometric validation in Syria was not performed. The survey also did not include potentially relevant factors such as physician age, seniority, communication style, clinical reputation, prior experience, physical appearance, or demeanor. Finally, multiple bivariate comparisons and domain-specific regression analyses were conducted without formal adjustment for multiple testing. These analyses were exploratory, and isolated statistically significant findings, especially those close to the 0.05 threshold, may reflect chance and should not be overinterpreted. In addition, the deviance goodness-of-fit test was significant for the psychological-issues model (p = 0.017), indicating possible model misfit despite the non-significant Pearson test; adjusted estimates from this model warrant particular caution. Sparse cells and wide confidence intervals in some models further limit precision.

### Future directions

Future research should explore how physician-sex preferences affect healthcare behavior and outcomes, including care-seeking, acceptance of examination, trust, adherence, and follow-up. It will also be important to examine whether other physician characteristics, such as experience, seniority, communication style, and clinical reputation, can reduce or override physician-sex preferences. Qualitative studies could clarify why participants prefer physicians of a specific sex in certain contexts and how these preferences are negotiated in real clinical encounters. Longitudinal or mixed-methods studies may help move the discussion beyond description toward evidence-based strategies that protect patient dignity while supporting equitable healthcare delivery.

## Conclusion

Preferences for physician sex among adults attending Syrian public teaching hospitals were context-dependent. Neutrality was common in several routine and non-intimate domains, but preferences became pronounced in sensitive contexts, especially intimate examinations, and varied by specialty. Women more often preferred female physicians for reproductive and intimate care, while men tended to prefer male physicians for general and intimate examinations. In exploratory adjusted models, participant sex was the most consistent predictor of preference, with education and religiosity showing domain-specific effects. These findings support offering targeted, context-specific options for sex-concordant care where feasible, while emphasizing communication, privacy, consent, and professionalism for all encounters. Service planning should reflect local patient comfort without reinforcing stereotypes or limiting professional opportunities. Future studies should assess whether accommodating preferences improves care-seeking, adherence, and outcomes, and should examine clinician attributes that may mitigate or amplify preference effects.

## Supporting information

S1 File Dataset and codebook. (XLSX)
